# Mind Your Own Business!

**Published:** 1918-10-26

**Authors:** 


### THE NURSING PROFESSION AND NURSES.

### "Mind Your Own Business!"

We are progressing! Nothing has pleased or interested me more in recent weeks than the Correspondence columns of The Hospital, despite the fact that most of the letters are hostile to my advocacy. What I like to see is that nurses and probationers alike are beginning to articulate. A mother's proudest moment is when she hears her babe's first word, and as word is added to word her delight is to tell her friends that "baby is beginning to speak." It means so much, this first indication of dawning intelligence. Therefore I loved to read those letters of criticism, and whether they are friendly or not matters little. When The Hospital entered on a campaign in favour of nurses' articulation none expected or hoped that the responding voices would be in unison. How dull would the world's Parliaments be if they were mutual admiration societies where each approved what the other suggested! I hold, and have always held, that criticism, free and bold, is the most wholesome atmosphere for the reformer. If his arguments are riddled by hostile fire, so much the worse for his arguments. They meet with the fate that they deserve. One cannot fail to respect the gunner who succeeds in silencing the enemy's artillery.

### The Letter of London Staff Nurses.

I am particularly interested in a letter which appeared in The Hospital on the 12th instant. It is signed by "Two Staff Nurses on behalf of others, London Hospital." It is a short letter, but it might have been even shorter. One line from the last sentence contains the essence of the whole—"We request Dr. Chapple to mind his own business." The same request is being made of me. Some there are who would describe this as rude. I do not. It is criticism, and as such commands attention. If I mistake not, Lord Knutsford meant much the same thing in this issue when he described Major Chapple, "of New Zealand," and all M.P.s as "honourable men." Major Chapple, presumably, is Brutus and Lord Knutsford Cæsar, not to say Kaiser. Let this pass. What I am concerned with is the "mind-your-own-business" attitude which, so far from being a petulant retort, represents and expresses a whole school of nurses and others engaged in hospital work. They resemble those creatures who have lived in the dark for so long that their optic nerves are atrophied, and they cannot see. In the long night of silence that has reigned in the world of nursing they have arrived at the erroneous conclusion that the British hospital is their exclusive possession, and that "outsiders" must be warned off the premises. "Mind your own business!" "Obviously an outsider" is "F. N.'s" description of "Ierne"!

### "Ierne" not Annoyed.

This, I may mention, does not annoy me in the least—nor does it amuse. I regard it as a medical man would regard a cough, or a pain, or an eruption. It is a symptom, and the disease that it suggests is either blindness, or ignorance, or both. Every British citizen, man or woman, perhaps especially the latter, is deeply concerned in the welfare of public hospitals. They are the refuge of the sick poor of the nation, founded and maintained by national subscriptions, and, so far from being trespassers or interlopers, public men and women who insist on holding aloft the lamp of truth and exploring these institutions are national benefactors.

### Is Major Chapple Spleenish?

May I inquire if there is any evidence that Major Chapple is animated by personal spleen? There may be; I do not know. But I suggest that if there is it ought to be produced. Most anxious appear some Hospital correspondents to make a personal attack on me, but never anxious to speak to the argument—also symptomatic of ignorance. I fail entirely to appreciate what interest it possesses for anybody, so far as the argument is concerned, whether Major Chapple likes or dislikes Lord Knutsford, nor even whether he comes from New Zealand, although I must confess I have a considerable regard for the Anzacs. Let us keep to the argument, and let it be clearly defined whose business is the British hospital, its nursing staff, and the well-being of its inmates. Whose? When this question is answered, those whom it may concern will realise and know how far the criticism "Mind your own business" is justified, and thus shall we arrive at a clearer vision.

### Nursing Ideals.

Now further. Much has been said by some Hospital correspondents concerning "the tenets and ideals of the nursing profession." I quote from "F. N.," and there are others. But be it observed that there is no attempt made to set out or define those tenets and ideals, nor, so far as I am aware, is there any code to which one can refer for the necessary information. "F. N.," referring to these, charges me with not knowing the meaning of the word "loyalty." I should like to judge myself, and I should also like others to judge me, but unless there is a standard this is impossible. I claim that I am loyal to my own ideals, but these may differ from other people's. And if it helps to clearer thinking and clearer expression I have no objection to set forth my own. I hold that the nursing of the nation's sick poor calls for the services of the best of the nation's women, and that the class of recruits that are now forthcoming are not the best. The hours of toil are too long, liberty too circumscribed, and remuneration far too poor to attract the best. I claim the House Governor of the "London" as my first witness. He openly states that nurses are shamefully underpaid. Is he minding his own business? Is he animated by spite? Is he "an outsider"? Is he among the loyal or the disloyal?

Let us above all things endeavour to be free from cant, and let the lamp of truth shine on the dark corners of the profession. The "mind-your-own-business" school live on cant and hypocrisy, and when they articulate, I, at least, am pleased. They talk in lofty measure, but their thoughts are mean, and they are void of argument. They claim a monopoly of highmindedness, of ideals, and resort to innuendo and abuse. I remember being struck with the wisdom of Lord Tennyson when he left the nation the imperishable heritage of his beautiful poetry. He saw with wonderful clearness of vision how carping critics would arise after his death and besmirch his memory by references of the Mark-Antony-honourable-man type, and he forbade the publication of his letters. "I do not want," he said, "to be ripped open like a pig"!

### The Argument—That's the Thing.

The argument!—that's the thing—always the argument, and only the argument. The rest is vulgar and unworthy of women of high ideals. British hospitals are a national possession, not the domain of an oligarchy. This newspaper for more than a generation has been a powerful factor in promoting their benefit and development. Their interest, the interest of the nurse, and the interest of the patient have been its constant concern. Who, in this crucial moment in the history of the British nurse, dare to say—"Mind your own business"? Come now, proud sisters, make your answer.

IERNE.

